# Remote Measurements of Tear Electrolyte Concentrations on Both Sides of an Inserted Contact Lens

**DOI:** 10.3390/chemosensors11080463

**Published:** 2023-08-17

**Authors:** Joseph R. Lakowicz, Ramachandram Badugu, Kundan Sivashanmugan, Albert Reece

**Affiliations:** 1 Center for Fluorescence Spectroscopy, Department of Biochemistry and Molecular Biology, University of Maryland School of Medicine, Baltimore, MD 21201, USA; 2 Department of Obstetrics, Gynecology and Reproductive Sciences, University of Maryland School of Medicine, 655 W. Baltimore St., Baltimore, MD 21201, USA

**Keywords:** contact lens, ion concentration, tear film, sodium-sensitive fluorophore, fluorescence sensing, tear composition, electrolytes in tears

## Abstract

In this paper, a method is described to perform ion concentration measurements on both sides of an inserted contact lens, without physical contact with the eye or the contact lens. The outer surface of an eye is covered with a tear film that has multiple layers. The central aqueous layer contains electrolytes and proteins. When a contact lens is inserted, it becomes localized in the central layer, which creates two layers known as the pre-lens tear film (PLTF) and the post-lens tear film (PoLTF). The PoLTF is in direct contact with the sensitive corneal epithelial cells which control electrolyte concentrations in tears. It is difficult to measure the overall electrolyte concentration in tears because of the small 7 μL volume of bulk tears. No methods are known, and no method has been proposed, to selectively measure the concentrations of electrolytes in the smaller volumes of the PLTF and the PoLTF. In this paper, we demonstrate the ability to localize fluorophores on each side of a contact lens without probe mixing or diffusion across the lens. We measured the concentration of sodium in the region of the PoLTF using a sodium-sensitive fluorophore positioned on the inner surface of a contact lens. The fluorescence measurements do not require physical contact and are mostly independent of eye motion and fluorophore concentration. The method is generic and can be combined with ion-sensitive fluorophores for the other electrolytes in tears. Instrumentation for non-contact measurements is likely to be inexpensive with modern opto-electronic devices. We expect these lenses to be used for measurements of other ions in the PLTF and the PoLTF, and thus become useful for both research and in the diagnosis of infections, keratitis and biomarkers for diseases.

## 1. Introduction

Contact lenses (CLs) are worn by 45 million Americans and over 140 million individuals worldwide [1,2] and must be regarded as a successful medical technology which allows an object to be placed directly on the eye. Most individuals adjust well to wearing CL. However, about 50% of first-time users discontinue use due to complaints of discomfort, dryness, and infections, such as keratitis, conjunctivitis (pink eye) and blepharitis infections of the eyelid [3,4]. Keratitis is a corneal infection that causes discomfort, vision loss, or blindness and may be caused by viral, bacterial, or fungal infections [5,6]. Prior to the 1970s, the incidence of keratitis was relatively rare [7–9], but with the large number of patients even a low incidence can be problematic.

CLs were commercially introduced in the 1970s [10,11], which was followed by an increased incidence of keratitis. Because keratitis is strongly associated with CLs use, there have been ongoing efforts for nearly fifty years to modify the properties of CLs and avoid keratitis. The cornea is the only non-vascular region in the body so corneal cells must obtain oxygen from the atmosphere [12]. Since the early CLs were not permeable to oxygen, a decreased oxygen supply seemed to be the cause of increased keratitis rates. This hypothesis resulted in intensive development of soft lenses made with hydrogels. This increased oxygen permeability but was not high enough for continuous wear of CLs. The known high oxygen diffusion and solubility in silicone [13,14] resulted in the development of silicone hydrogel (SiHG) lenses with dramatically increased oxygen transport rates, which could be higher than an equivalent thickness of water [15,16], and allowed the lenses to be worn for days or even while sleeping [17–19].

In spite of these developments, the incidence of keratitis remained high and were even found to be higher with soft lenses rather than hard lenses [5–7]. These results suggest that corneal changes and infections are the result of physical presence of the lens directly over the epithelial layer of the cornea, and not the chemical composition of the CL.

**Figure 1.**
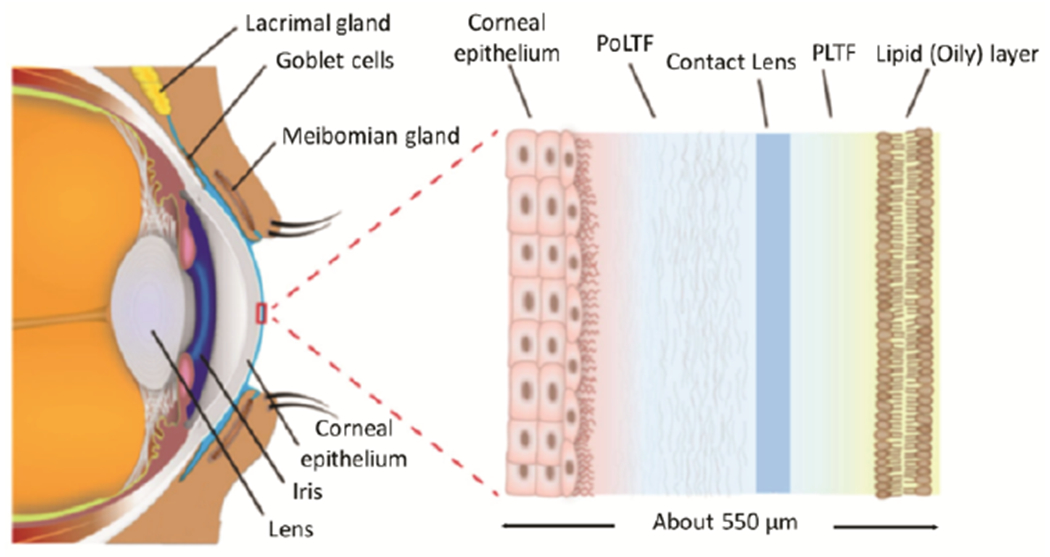
Structure of the cornea with an inserted contact lens (darker blue), floating in the central aqueous region (lighter blue) revised from [20].

Composition of tear films. The outer surface of the eye is protected by a thin tear film [20]. Tear films have a multi-layer structure, as illustrated in Figure 1, and the layers have distinct characteristics and functions. The outermost layer of tear film is the first surface encountered by airborne contaminants such as dust, bacteria, or viruses. Contaminants are rapidly swept across the eye by the eyelid for disposal into the nasolacrimal duct and it is therefore difficult to know the site of origin of eye infections. This outer top layer is rich in lipids which reduce water evaporation from the middle aqueous layer. But evaporation cannot be avoided, which is why we blink 15 to 20 times per minute. The tears are continually replaced by secretions from the lacrimal glands, and by the meibomian glands which secrete lipids, at a rate of 35% replacement per minute [20,21], The tear film forms spontaneously from these secretions.

**Figure 2.**
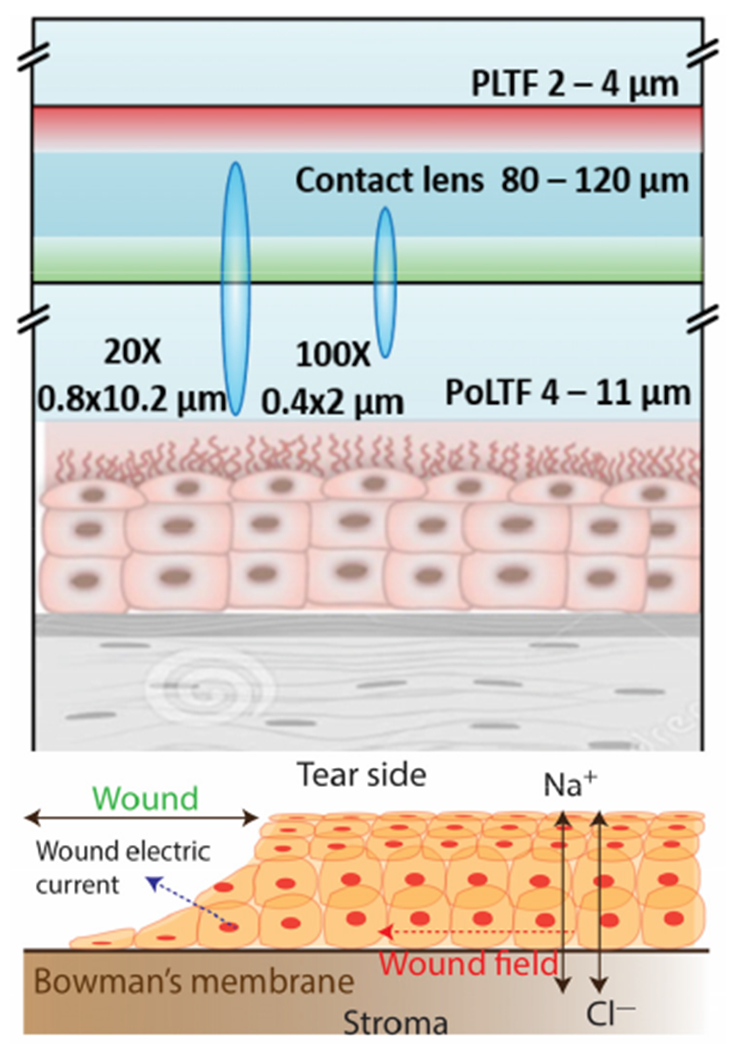
Top, expanded view of the cornea, contact lens and tear films. Green regions are surface-localized fluorophores at the PoLTF. The red region indicates fluorophores at the PLTF. The light blue ellipticals show the diffraction limited observation volumes with 20× and 100 × objectives. The lower panel shows the wound ion currents.

The central layer of a tear film is aqueous and contains a variety of proteins, antibodies and electrolytes needed for the bottom layer of corneal epithelial cells. Prior to insertion of a CL, there is a single continuous central aqueous layer. When a CL is inserted, it becomes localized in the central aqueous region which creates two new tear layers called the pre-lens thin film (PLTF) and the post-lens tear film (PoLTF) (Figure 1). The thickness of the PLTF and the PoLTF are not known precisely and are likely to vary with physiological conditions [22–27]. Methods to measure thickness of these layers include in vivo confocal microscopy (ICM), optical correlation tomography (OCT) and interferometry [27–30]. Typical reported thicknesses are 2–4 μm and 4–11 μm for the PLTF and the PoLTF, respectively, as illustrated in Figure 2 [31–34].

The bottom layer of epithelial cells is about 7 cells thick and have a lifetime of 7 to 10 days. These cells are active in ion transport [35–38] to maintain the electrolyte balance in tears (Figure 2). Numerous publications have reported on the total ionic composition of tears. All these papers report on the ion concentrations of bulk tears, do not separate the volumes of the PLTF and the PoLTF and do not provide measurements of the individual electrolytes. It is easy to imagine the ion concentrations can be different in the PLTF and the PoLTF, especially if the lenses are not permeable to ions and the epithelial cells are active in ion transport. Measurements of the individual electrolyte concentrations are not reported because of the difficulties in sampling tear fluid. The fluid volume in a single eye is near 7 μL, and the eyes respond rapidly upon any contact resulting in increased secretion of the lacrimal glands which changes ion concentrations [39–42]. At present, there are no methods to selectively obtain tear samples from the PLTF and PoLTF regions. The samples are collected from the cornea of the eye, not near the CL. The most common measurements in current use depend on briefly touching the eye to collect a sample with basal ion levels obtained before contact-induced changes in ion concentrations. The total electrolyte concentration is calculated only by measurement of the total conductivity. Because of this limitation, there are few reports which demonstrate a relationship between individual ion concentration and ocular pathology. Smart contact lenses to measure electrolytes, glucose and other analytes in tears have been reported, but the measurements require complex metal components and sensing components to be built within the contact lens [43–46].

Contact Lens Development. Modern CLs were introduced in 1961 and the first full-scale commercial introduction was in 1971 [10,11]. The first lenses were rigid and made of glass or poly(methyl methacrylate) (PMMA). While initially successful for improved vision, they were not suitable for long-term wear because they are not permeable to oxygen or ions. Corneal cells do not have a vascular source for oxygen and can obtain oxygen only from the air. Soft CLs were developed for allowing increased oxygen and ions flows across the lens, and to provide flexibility. The soft lenses are made of hydrogels (HG) formulated with various compositions of carbon and oxygen-containing monomers. However, the HG lenses were not adequately permeable to oxygen and were not recommended for continuous wear or while sleeping. Attempts were made to increase the oxygen permeability by decreasing HG polymer content, but the HG lenses become too frail for practical use with this decrease.

**Figure 3.**
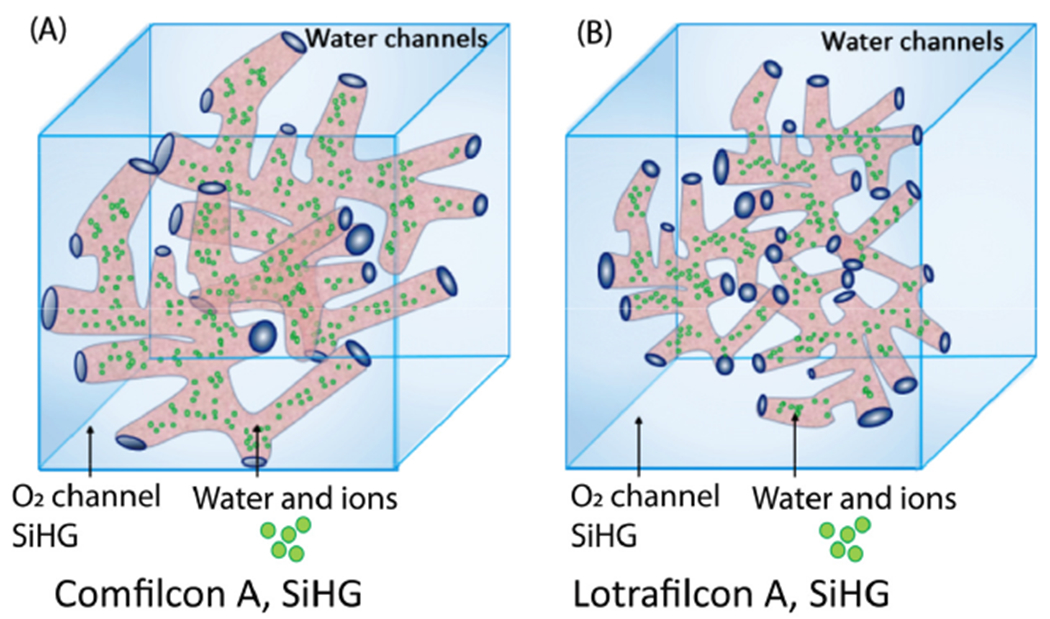
(**A,B**) Schematic representation for interpenetrating polymer networks for contact lenses with high or low silicone content. Light blue indicates silicone regions and light pink represents the water or tear fluid channels. The green dots show the locations of the ionic species in the lenses. Comfilcon A probably has larger water channels as a result of the higher water content.

Silicone was known to be highly permeable to oxygen due to rapid diffusion and high oxygen solubility [13,14], which motivated the development of SiHG CL. But silicone is hydrophobic and does not mix with water. The initial SiHG lenses had hydrophobic surfaces which resulted in discomfort and damage to the tear film [22–24]. The surface hydrophobicity was decreased with various surface treatments. It took about 20 years of research by multiple companies to develop optically clear SiHG lenses with hydrophobic surfaces which provided both high oxygen and ion permeabilities. The SiHG monomers typically contain silicone regions and regions with carbon-hydrogen polymers groups for crosslinking. The important structural aspect of SiHG lenses is the presence of continual aqueous channels from the front to the back of the lens for tear transport, and regions of nearly pure silicone for oxygen transport (Figure 3). SiHG lenses consist of interconnected hydrophobic, non-polar silicone areas and polar regions that contain water or tear fluid covering the entire lens. These kinds of structures are often referred to as interpenetrating polymer networks (IPNs). The IPN interface region offers a place to attach modified ion-specific fluorophores with moieties, such as poly-L-lysine. The fluorescent probe stays within the aqueous channels and can come into contact with electrolytes of interest [47,48]. On the other hand, the aqueous region of SiHG is called a semi-IPN. There is a complex nomenclature to describe such structures. There are different types of IPN structures, but we will refer to these as IPNs. This was a remarkable accomplishment for chemists to obtain an IPN with a channel diameter smaller than the wavelength of light which resulted in clear lenses and did not scatter light. Some lenses could be worn for 30 days even while sleeping [25,26]. Because of continued infections (discussed below), SiHG lenses were made with a lower silicone content (Table 1). Many of the problems with lenses such as stiffness, hydrophobicity and low oxygen transport were solved with this continued refinement of CL polymers and their surfaces. However, the incidence of keratitis has remained constant or even increased for some SiHG lenses. This increase was a surprise because the increased oxygen permeability of SiHG lenses was expected to decrease the incidence of keratitis. However, the oxygen permeabilities were not adequate to supply oxygen to the cornea and continuous wearing of CL was not recommended. The introduction of SiHG lenses resulted in softer lenses that displayed oxygen permeability even higher than an equivalent thickness of water. Unfortunately, the new softer HG and more permeable SiHG lenses did not result in a decreased incidence of keratitis. The incidence does not appear to be linked to the hardness, chemical composition, or oxygen permeabilities of the different lenses. The persistent incidence of keratitis suggests that infections are the result of physical presence of the lens directly over the epithelial layer of the cornea, and not the chemical composition of the lenses.

In the present paper, we describe the use of fluorophores that can bind selectively to one side of a CL. Surface-selective binding provides an opportunity to measure ion concentrations in the PLTF and the PoLTF. Depending on the lens and tear exchange in the PoLTF, the ion concentration will be useful in fundamental research, testing new CL and in diagnosis of ocular pathologies.

## 2. Materials and Methods

The fluorescence probe sodium green (SG, tetramethylammonium salt, cell impermeant) was obtained from Thermo Fisher Scientific, Stoney Creek, CA, USA. The other two probes, fluorescein (FL) and rhodamine B (RhB), were purchased from Sigma-Aldrich, St. Louis, MO, USA. Other chemicals included N-(3-dimethylaminopropyl)-N’-ethylcarbodiimide hydrochloride (EDC), N-hydroxysuccinimide (NHS), and poly-L-lysine (PL) (150–300 kDa). Ultrapure water (with a resistivity of 18.2MΩ-cm) purified using aMilliporeMilli-Q gradient system was used in the preparation of aqueous solutions.

Synthesis of poly-L-lysine derivatives of fluorescein (FL-PL), rhodamine (RhB-PL) and sodium green (SG-PL). Fluorescein and RhB each contain a single free carboxyl group which was linked to PL using known coupling procedures. PL was selected because of its high affinity for SiHG lenses. The chemical synthesis and testing of SG-PL for sodium sensing was described previously. The commercially available SG is a dimer of two fluorescein-like aromatic rings linked by a semi-rigid organic linker. The chemical structure of the commercially available SG is shown in Scheme S1 in Supplementary Materials. The fluorescent rings differ from fluorescein by the addition of a chloride atom on each end. Because of the SG dimeric structure, there are two free carboxyl groups; both are used for conjugation with PL. The emission spectrum of SG-PL (Figure S1) is very closely matched to that of FL-PL. The SG-PL was prepared following activation of the free carboxyl groups on SG, and subsequent amide bond formation with PL. An aqueous solution of 0.01% PL (MW 150–300 kDa, 0.45 mL) was added to the solution of SG (1 mg, 6.0 × 10^−4^ mmol) in 2 mL of dimethylformamide (DMF), which was activated with N-(3-dimethylaminopropyl)-N’-ethylcarbodiimide hydrochloride (EDC, 0.34 mg, 1.8 × 10^−2^ mmol) and N-hydroxysuccinimide (NHS, 0.21 mg, 1.8 × 10^−2^ mmol); see reaction Scheme S1 in Supplementary Materials. For the preparation of FL-PL and RhB-PL, we used FL (0.2 mg, 6.0 × 10^−4^ mmol) and RhB (0.29 mg, 6.0 × 10^−4^ mmol) instead of SG. The reaction solutions were gently agitated overnight at room temperature under an inert atmosphere. Subsequently, the poly-L-lysine-probe conjugates were purified via dialysis against MOPS buffer (pH 7.2, 20 mM) using an 8 kDa molecular weight cutoff dialysis cassette (Pierce, CO, USA).

Side-specific labeling of contact lenses. CLs were prepared for labeling by first washing with water to remove the storage buffers and preservative materials. For side-specific labeling, the CL, with the corneal side focusing upwards, was placed into a 20 mm Petri dish with 0.3 mL of the desired probe solution (FL-PL or RhB-PL), and the incubation continued for 30 min. For double labeling, 0.2 mL of the second probe solution was placed onto the upward facing side of the lens, again followed by 30 min incubation. The labeled lenses were extensively washed with deionized water before being used for experiments. For emission studies (non-confocal), the lenses were held diagonally in a 1 cm × 1 cm cuvette and covered with the appropriate buffer and salt concentrations. Unless stated otherwise, all experiments were performed in 20 mM MOPS buffer, 8 mM potassium ion concentration, pH 7.2, and at room temperature.

Selection of contact lenses. The area of contact lens polymer chemistry is continuously progressing, resulting in the creation of lenses that now contain only small quantities of silicone. To implement these modifications, we previously developed a sodium-sensitive fluorophore that is linked to PL. PL could bind to the non-polar regions of SiHG. SiHG polymers are made using monomers that contain regions with partially oxidized carbon atoms, carboxyl groups, and cross-linkers. These groups have the ability to impart a negative charge to the lens and facilitate the electrostatic binding to PL, as described in the reference [47,48].

Numerous SiHG lenses are offered for sale by multiple companies. We recognized that different surface treatments by different companies could affect probe binding and surface localization. We selected two widely used lenses with different silicone contents (Table 1). Lotrafilcon A (LotA) has 24% water versus 48% water in Comfilicon A (ComA), with variable correction factors. The central thickness of Com and LotA is about 80 ¼m. It appears that the ComA lenses were designed to have a higher water content for patient comfort. These dimensional structures are most probably random and images of the IPN are not available. We reasoned that the aqueous channels in ComA could be larger than LotA due to the higher water content of ComA, as illustrated in Figure 3. This is our speculation and is not supported by published data.

Fluorescence Measurements. The fluorescence spectra and intensity decays were measured using a Varian Cary Eclipse 4 spectrofluorometer and a FluoTime 300 instrument from PicoQuant (Berlin, Germany), respectively. A 495 nm excitation source from a Solea supercontinuum laser and a 473 nm pulsed laser diode with a repetition rate of 40 MHz each served as the excitation sources. The emission spectra and intensity data were an average of three measurements, and they were analyzed using the EasyTau program from PicoQuant. Fluorescence lifetime imaging microscopy (FLIM) was conducted utilizing a laser scanning confocal microscope from ISS (Champaign, IL, USA), which included dual scanning capacity for Z-scan intensity measurements of the CL. For the FLIM experiments, a 473 nm wavelength pulsed laser diode source was used. The emitted light was seen through a 575 nm filter, which contained a bandwidth of 105 nm. Additionally, a 20× objective and a 25 μm pinhole were used in the confocal experiments. A mechanical stage scanner was used to capture images of the complete CL, with an image size of 1 cm × 1 cm and a resolution of 256 × 256 pixels. The galvo scanning mirror was also used to image small area with an image size of 250 μm × 250 μm and a resolution of 256 μ 256 pixels. The FLIM images were subjected to pixel-wise analysis using a two-component decay analysis model. The darker blue ellipses in Figure 2 show the theoretical dimensions of the confocal incident light or the observed confocal volume using respective objectives. The Z-axis resolution is expected to be adequate for selective detection from each side of the lens.

## 3. Results

**Figure 4.**
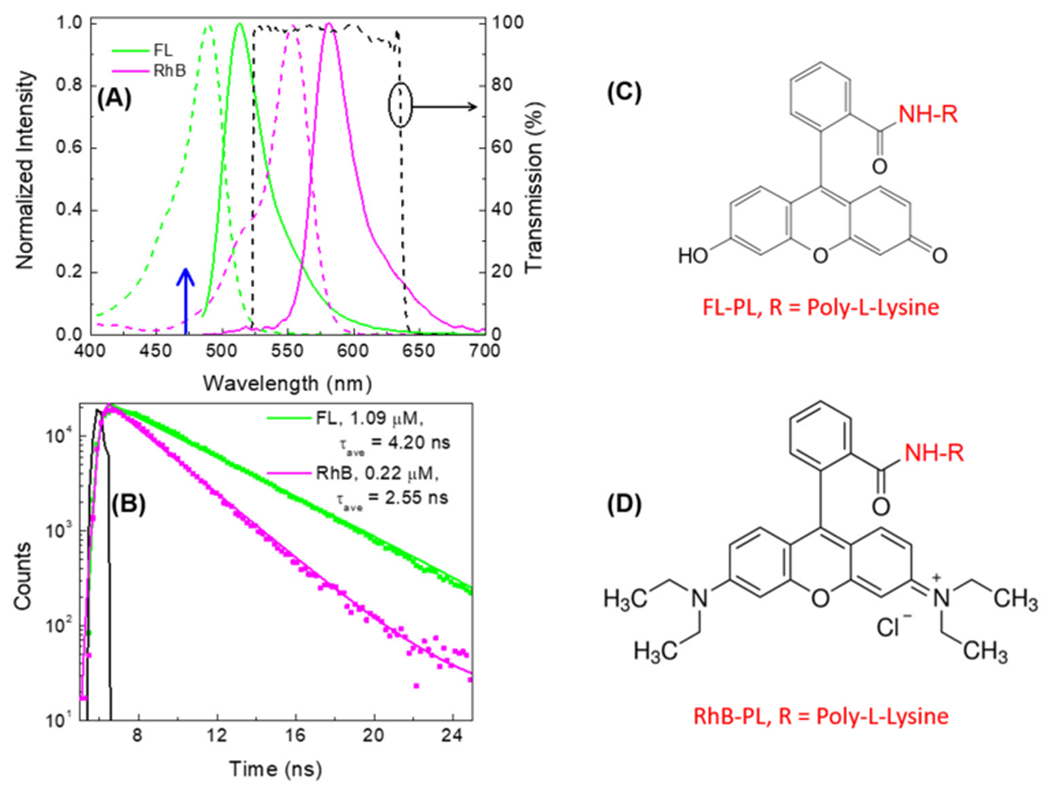
Fluorophores (FL and RhB) were selected for side-selective labeling of contact lens. (**A**) Normalized excitation (dashed line) and emission (solid line) spectra and (**B**) intensity decays of FL and RhB in 7.2 phosphate buffer. Also shown in the figure is the transmission spectra of the emission band-pass filter (575/105 nm) used for the present study. The black line is the impulse response function. The concentrations of FL and RhB were 1.09 and 0.22 μM, respectively. (**C,D**) Chemical structures of FL-PL and RhB-PL with the polylysine groups shown in red. The bold blue arrow in panel A is the selected excitation wavelength for both probes.

Measurement of side-selective detection. Fluorophores, FL and RhB, were selected for the side-specific labeling (SSL) experiments because they could be observed at different emission maxima and different intensity decay times. Both probes could be excited at 473 nm (blue arrow, Figure 4A). Figure S2 shows the un-normalized emission spectra at the same probe concentrations. The absorption coefficients at 473 nm are similar, but FL displays an approximately 5-fold higher peak intensity. The weaker RhB emission was found to be favorable by allowing for more direct detection of the sodium sensitivity probe Sodium-Green-PL.

**Figure 5.**
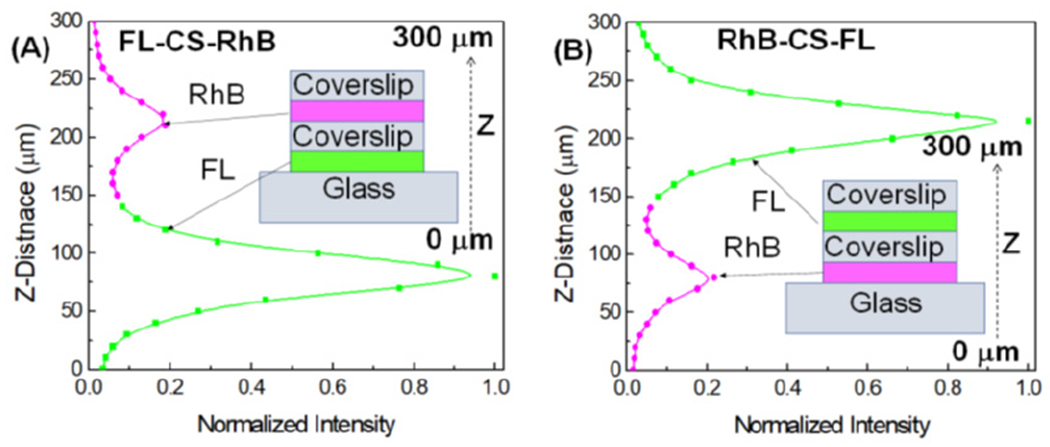
Confocal fluorescence intensities measured along the Z-axis for structures of (**A**) FL-RhB and **(B)** RhB-FL in two different locations separated by a cover slip (CS). The observed 170 μm distance between the intensity peaks are consistent with the 170 μm thickness of cover slip.

Confocal fluorescence microscopy was used to independently detect the two probes separated by a glass cover slip about the thickness of a contact lens (Figure 5). The same emission filter was used to detect both probes. The confocal intensities were measured along the vertical Z-axis, across the probe layers and cover slips (Figure 5A,B). In these figures, the confocal plane is plotted along the Z-axis, same as the detector motion. The emission intensities are plotted along the X-axis. Two distinct intensities peaks were observed along the Z-axis (Figure 5). The changing color of the data line is to indicate the different probes emission which passed through the 575/105 nm emission filter which transmits emission from both probes (Figure 4). Independent of the top or bottom location of the probes, the FL intensity is brighter than the RhB intensity. This result is consistent with Figure S2 and also shows that the intensities in Figure 5 are not affected by self-absorption or re-absorption of emission. The Z-distance between the FL and RhB emission maxima is 170 μm, which is in precise agreement with the known cover slip thickness. The Z-distance width of the intensities is about 40 μm, several-fold larger than the resolution-limited distance of 10 μm with a 20× objective (Figure 2). The increased width can be due to many reasons, including the multiple surfaces and refractive index differences in the glass and water layers. The results demonstrate that even with decreased Z-resolution a confocal microscope can selectively detect fluorophores across the thickness of a CL.

The identity of the fluorophores on each side of the cover slip was verified using confocal fluorescence lifetime imaging microscopy (FLIM) [49,50], which were found to be in agreement with Figures 4 and 5. The confocal intensity images (Figure S3A,C) were uniform across the field-of-view (FoV) showing that the probes on each surface were not aggregated and were uniformly distributed over the lens surfaces. The lifetime of RhB of 1.9 ns and Fl of 3.8 (Figure S3E) are in good agreement with the values measured in cuvettes (Figure 4). The spatial accuracy of FLIM is seen in the lifetime histograms constructed from all the pixels in the image (Figure S3E). We were surprised that the minor side peaks in Figure 5 did not appear in these histograms. The FLIM instrument displayed good accuracy even at the single pixel level with values ranging from 1.6 to 2.4 ns for RhB and from 3.0 to 6.4 for FL (Figure S3F).

**Figure 6.**
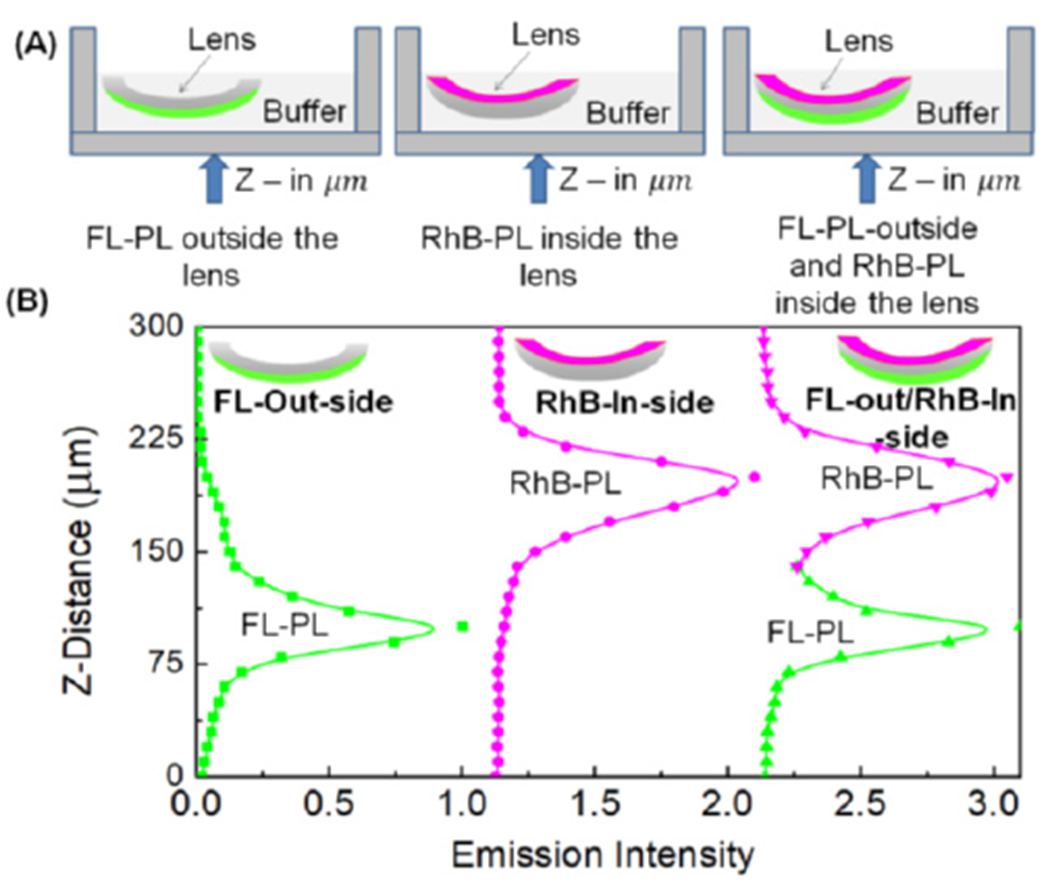
(**A**) Schematic of lens labeling procedure. (**B**) Z-axis intensity scans of FL-PL outside-labeled Lotrafilcon A lens, RhB-PL inside-labeled lens and FL-PL and RhB-PL double-labeled lens. λex 473 nm, 575/105 emission band-pass filter, 25 μm pin hole, 20× objective. Green and pink lenses and lines, respectively, for FL-PL and RhB-PL.

Side-selective labeling of contact lenses. The ability to measure each side of a CL was tested with FL-PL and RhB-PL. Interpretation of the results was simplified because the decay times were not sensitive to ions, such as sodium. These probes bound rapidly and non-reversibly to Lotrafilcon A lenses. The separate surfaces were labeled by floating the lens in buffer and adding probe solution into the desired surfaces (see Section 2). The surface selectivity (Figure 6) did not display extra peaks and was selectively superior to the glass slide in Figure 5. The probe intensity peaks were separated by 70 nm, in good agreement for a Lotrafilcon A lens. The width between the intensity peaks was also reduced when compared to the cover slip measurements. The absence of additional intensity peaks along the Z-axis indicates that no detectable amount of either probe diffused crossed the Lotrafilcon A lenses. Similar results were observed previously for surface-bound proteins but have not been reported for smaller organic probes.

**Figure 7.**
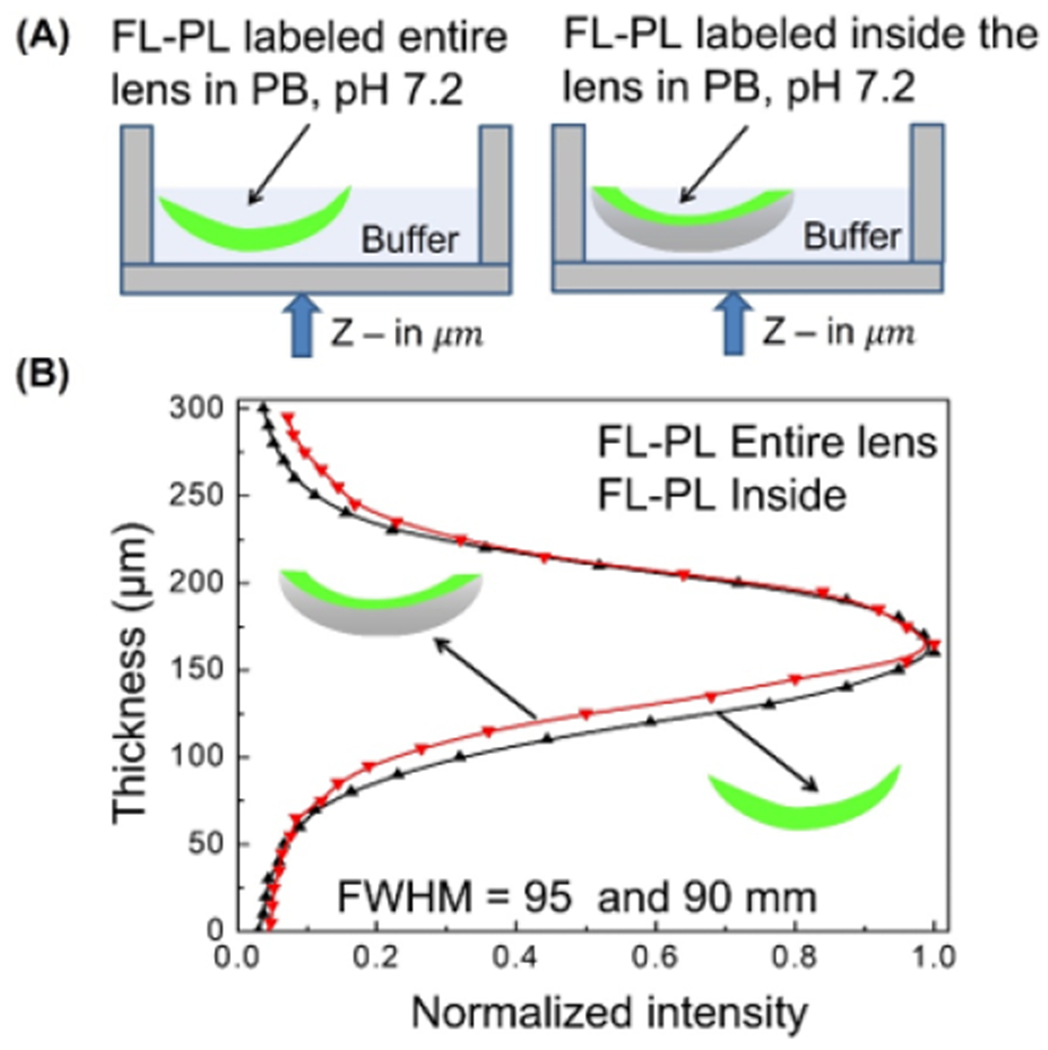
(**A**) Schematic of lens labeling procedure for Comfilcon A with FL-PL. (**B**) Z-scan of emission intensity distribution of FL-PL which labeled the entire and inside the Comfilcon A lenses. λex = 473 nm, 575/105 nm band-pass emission filter, 25 μm pinhole, 256 × 256 pixel resolution, 450 × 450 μm image size, 20× objective.

We questioned if surface-selected labeling would occur for other SiHG lenses. This possibility was tested with Comfilcon A lenses, which were labeled with FL-PL (Figure 7). Irrespective of the labeling procedures, the FL-PL did not localize at the surface and was evenly distributed across the lens (Figure 7). The width of the labeled peak was near 95 nm, which is consistent with the Z-resolution of our confocal microscope and the thickness of Comfilcon A lens. Similar results were obtained with RhB-PL-labeled lenses (Figure S4). The results in Figures 6 and 7 indicated that surface localization of fluorophores is not a general phenomenon for all contact lenses. Surface localization must be tested with each type of contact lens used for side-specific sensing.

**Figure 8.**
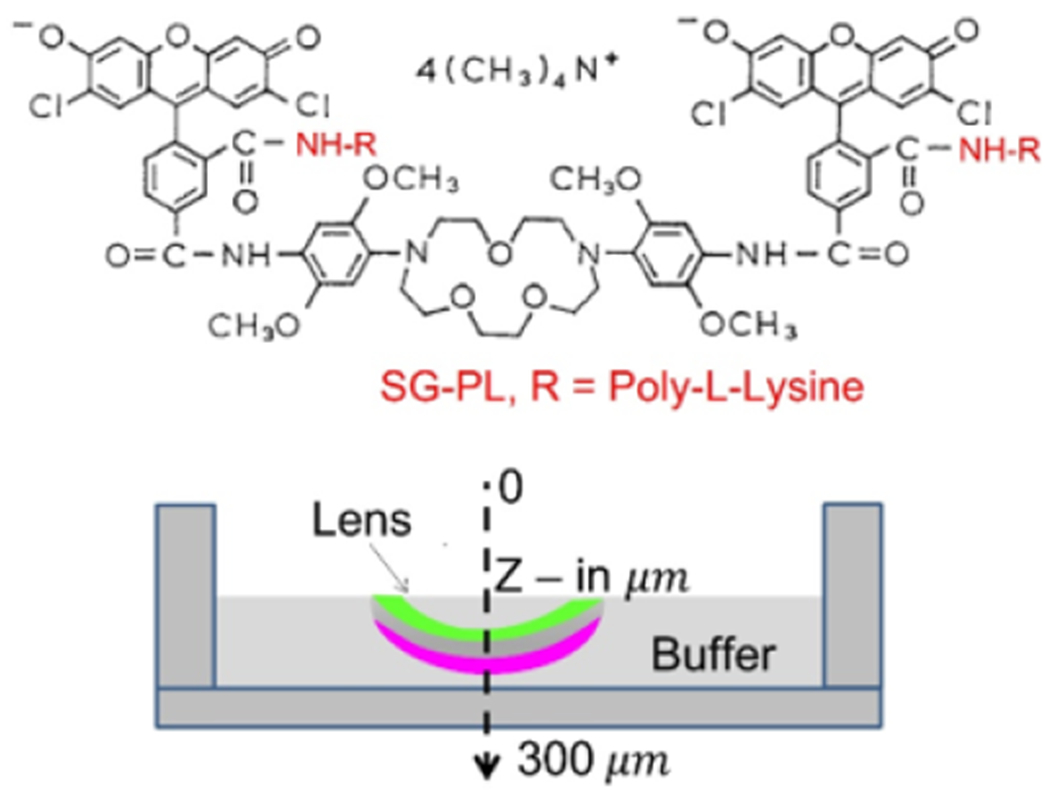
Chemical structure of SG-PL (**top**). Schematic of the RhB-PL and SG-PL double-labeled Lotrafilcon A lens and Z-scan measurement direction (**bottom**).

**Figure 9.**
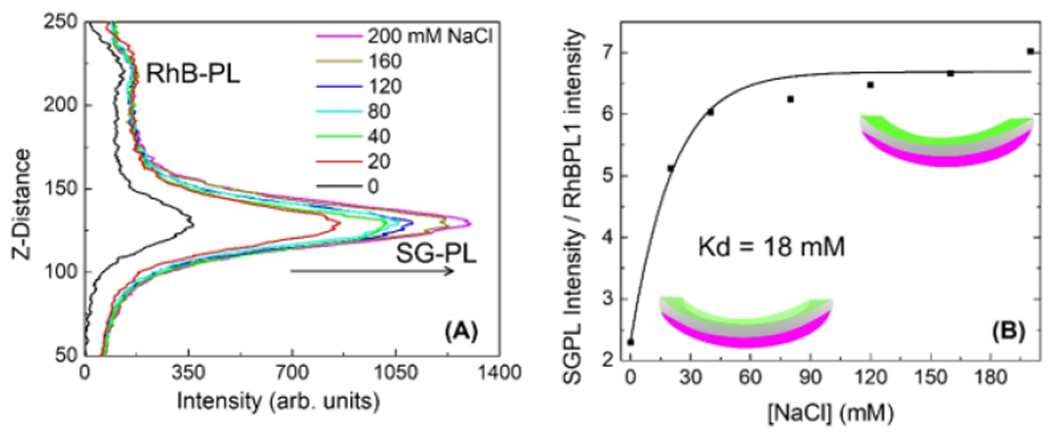
(**A**) Effect of Na^+^ concentration on the Z-scan intensity on RhB-PL labeled outside and SG-PL labeled inside of the Lotrafilcon A lens in MOPS buffer (20 mM). (**B**) Intensity ratio from the SG-PL region to that of RhB-PL region in a double-labeled lens with respect to Na^+^ concentration. Insert, Green and pink color, respectively, for FL-PL and RhB-PL.

Sodium sensing on the inner surface of a Lotrafilcon A CL. We previously described a sodium-sensitive fluorophore which binds to three different SiHG lenses [42–48,51]. To determine if surface-sensitive sodium detection was possible, we labeled a Lotrafilcon A lens with SG-PL on the inner surface (Figure 8). The outer surface was labeled with RhB-PL, which is not sensitive to sodium, and thus serves as a reference for wavelength-ratiometric sensing. The SG-PL intensity increased about 8-fold in response to Na^+^, while the RhB-PL intensity remained constant (Figure 9). The lens with this combination of fluorophores was useful as wavelength-ratiometric probe for Na^+^ with a Kd = 18 mM (Figure 9B), in agreement with our previous reports [47–49,52].

If the lens were to be placed on an eye, it would be able to indicate the concentration of Na^+^ in the PoLTF. The described lens could also be used with FLIM on lifetime-based sensing, but the decay times are more closely spaced than FL-PL and RhB-PL which may be the result of overlapping emission. Although the leakage of probes from the lens and their diffusion into the lens mostly rely on the concentration of SG-PL used in the lens doping, thus far, we have not seen any probe leaking from the lens. However, over time, the small amount of probes may diffuse into the interior space of the lens owing to its porous structure. Additionally, we previously noticed minimum lateral diffusion of SG-PL using several kinds of contact lenses [48].

## 4. Discussion

The field of contact-free remote sensors of ion concentrations in tears in both the PLTF and the PoLTF is in its infancy. The previous results and publications [47,48] have answered many questions about the feasibility of ion-sensitive contact lenses. Contact lenses with bound fluorescent ion sensors are expected to play an increasing role in research and clinical testing in ophthalmology. At present, there are only a few clinical tests used by ophthalmologists for diagnosis of dry eye disease (DED). The dominant ones are the Schreiner test for dry eye by the rate of tear production using a paper strip in the eye. Tear production is also measured from the tear break up times (TBUTs) using eye drops containing dyes like fluorescein and watching the fluorescein film until it breaks up [53]. A more recent test takes a rapid-touch sample of tears and measures the total electrolyte concentration [54–56]. DED is known to be associated with an altered total ion concentration in tears. We expect the label CL will replace these older tests. This change in clinical practice will require considerable additional research in the areas of probe synthesis, selection of suitable CL polymers, and in vivo testing. Many ion-selective fluorophores are known after the chemical structures have been modified to obtain the desired ion binding content in the measurement location.

Our approach to fluorescence sensing CL avoids the problems encountered in sensing lenses with built-in electronics for electrochemical or colorimetric detection. Such lenses are expensive to fabricate and are not likely to be disposable with lenses which are discarded after one day of use. Also, electronics can block a part of the vision field. Our low-cost fluorescence sensing contact lenses (FS-CL) will provide the advantages of an optical sensor that is not in direct contact with the eye. Also, the FS-CL will not require a power supply in the lens or RF energy by inductance. The initial use of FS-CL will not be for continuous measurement over hours or days. In a doctor’s office, the lenses will be inserted into the patient’s eye and a return to basal level tears occurring in less than 15 min will be measured.

An advantage of side-selective labeling of CL is the ability to measure ion concentration on both sides of a CL in the PLTF and the PoLTF. At present, no ion concentration measurements have been reported within these films, but there are multiple reasons why the ion concentrations may be significant. A lens on the cornea is mostly stationary in the PoLTF, which may decrease the tear exchange rate, compared to six times per minute exchange rate in the PLTF. An infection of the eyelid may change the ion concentrations in the PLTF, or an infection of the cornea may change the ionic composition of the PoLTF. If such changes occur, the duration of the differences will depend on the rate of PLTF-PoLTF exchange and/or the permeability of the lenses to ions. The results in Figure 9 suggest another use of side-specific labeling. The non-response RhB-PL can be used as a reference intensity for the response of other ion-sensitive fluorophore. The thickness of the lens results in a distance that is too long for fluorescence resonance energy transfer (FRET) to occur. FRET could occur if both fluorophores were on the same side of a contact lens.

The measurements with fluorescent CL do not require eye contact and are mostly independent of eye motion and fluorophore concentration [49,50]. The method is generic and the many ion-sensitive fluorophores can be applied to all electrolytes in tears. Instrumentation for non-contact measurements is likely to be inexpensive with modern electronic devices and solid-state CMOS detectors. The results in the present paper are aligned with two emerging trends in research. One rapidly advancing trend is the development of CL which contains electronics and sensors for specific analytes [56–58]. Given the rapid progress of smaller electronic components, we expect to see an increasing number, and even some with visual displays. These hybrid electronic devices have two disadvantages. First, the visual field may be partially blocked by the electronic components. A second more difficult challenge is the continuing migration to CL which are used for only one day. Such lenses are often called daily disposable (DD). Use of DD lens allows to avoid the inconvenience of daily cleaning lenses and the increased risk of infection. This goal will require additional synthesis and testing of ISF for many different CLs. After performing a brief search on the internet with a significant number of CL, it became clear that it would be challenging to cut down the cost to that of DD lenses.

Another factor is the availability of tears as an alternative to blood samples. Many, if not most biomarkers, in the blood can also be found in tears [5]. In the future, it seems likely that CL coated with antibodies will be used to detect biomarkers [59–61]. These biomarkers may include those known for cancer and for neurodegenerative diseases [62–64].

It is our opinion that fluorescent CL will remain less costly than electronic lenses. Over the next two decades, we expect sensing lenses based on fluorescence to become a widely used technology.

## Supplementary Material

SM

## Figures and Tables

**Table 1.** Silicon and water content of silicone hydrogel contact lenses were used for the present study.

Polymer	Trade Name	Manufacture	Wear Days	Water %	Dk (at −3.00 D) *
Lotrafilcon A (SiHG) ^1^	Air Optix Night and Day Aqua	Alcon	30	24	175
Comfilcon A (SiHG) ^2^	Biofinity Multifocal	Cooper Vision	30	48	160

1 https://professional.myalcon.com/contact-lenses/monthly/air-optix-night-and-day/ (accessed on 1 May 2023).

2 https://coopervision.com/sites/coopervision.com/files/product-specs/coopervision-product-specifications-us-1121.pdf (accessed on 1 May 2023).

* Dk is the product of the diffusion coefficient and the oxygen permeability.

## Data Availability

Researchers can obtain the date from the corresponding author upon request.
