# Supplementary material for: Remote Measurements of Tear Electrolyte Concentrations on Both Sides of an Inserted Contact Lens
Source: Chemosensors (Basel). Author manuscript; Available in PMC 2024 Jan 25. (PMC10810336; doi:10.3390/chemosensors11080463)
Supplement: SM [file NIHMS1955891-supplement-SM.pdf]

chemosensors

MDPI

Supplementary Materials

Remote Measurements of Tear Electrolyte Concentrations on Both Sides of an Inserted Contact Lens

Joseph R. Lakowicz [1,*], Ramachandram Badugu [1], Kundan Sivashanmugan [1] and Albert Reece [1,2]

1 Center for Fluorescence Spectroscopy, Department of Biochemistry and Molecular Biology, University of Maryland School of Medicine, Baltimore, MD 21201, USA; rbadugu@som.umaryland.edu (R.B.); skundan@som.umaryland.edu (K.S.); areece@som.umaryland.edu (A.R.)
2 Department of Obstetrics, Gynecology and Reproductive Sciences, University of Maryland School of Medicine, 655 W. Baltimore St., Baltimore, MD 21201, USA
* Correspondence: jlakowicz@som.umaryland.edu

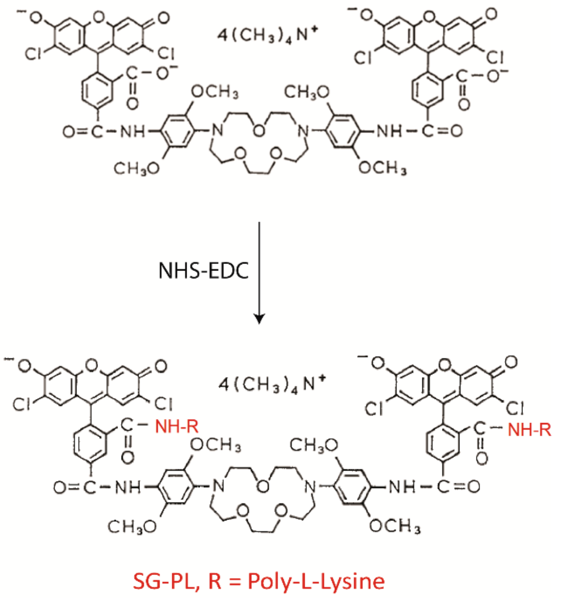

Scheme S1. Chemical synthesis and structures of sodium probes for use in CL.

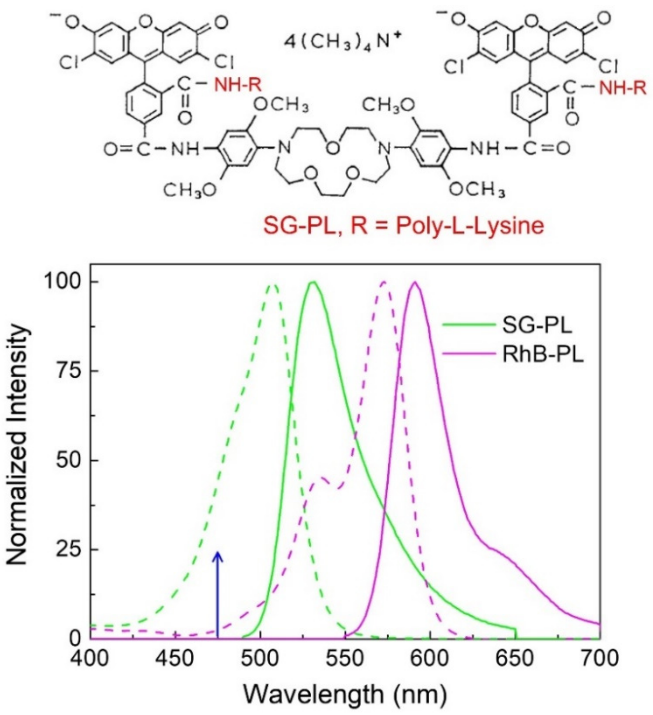

Figure S1. Chemical structure of SG-PL (top). Normalized excitation (dashed line) and emission (solid line) spectra SG-PL and RhB-PL in 7.2 phosphate buffer.

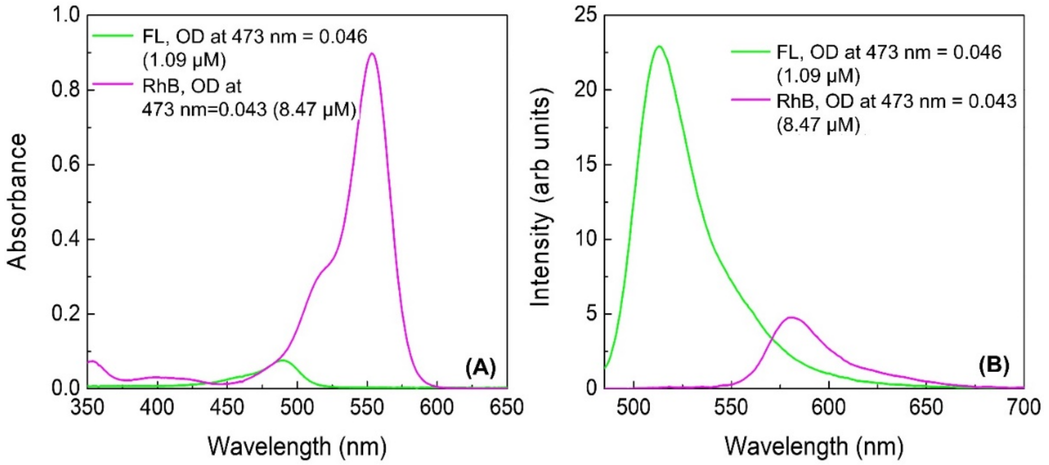

Figure S2. (A), Absorption and (B) emission spectra of fluorescien (FL) and rhodamine B (RhB) in pH 7.2 phosphate buffer. Iso-OD solutions at 473 nm with concentrations of 1.09 and 8.47 μM for FL and RhB, respectively, were used. λex = 473 nm.

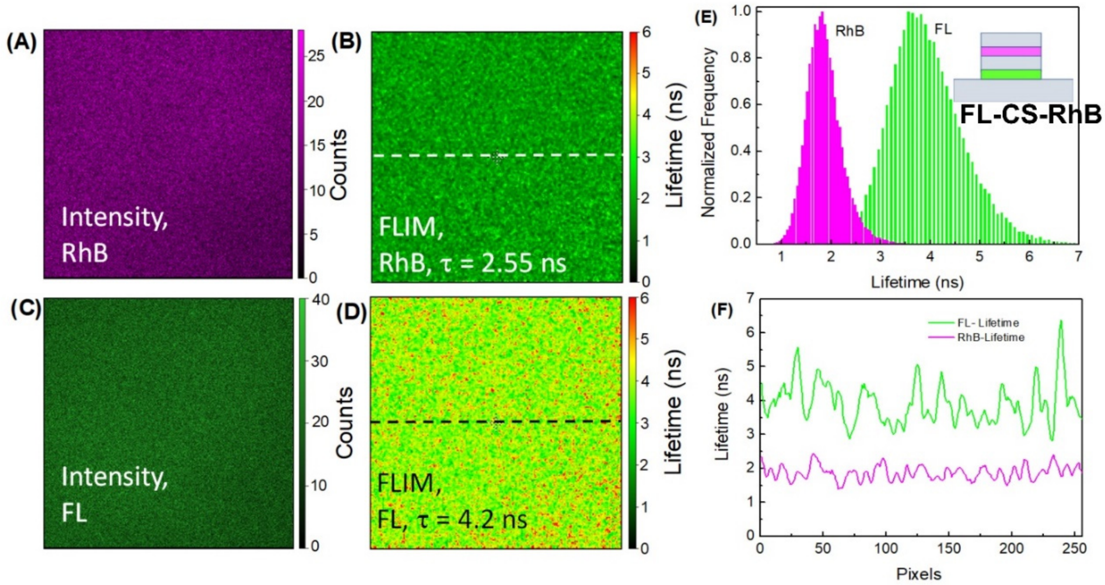

Figure S3. Confocal intensity images (A and C) and FLIM images (B and D) from RhB-layer and FL-layer, respectively, in FL-CS-RhB system shown. Intensity images were color coded with green for FL and magent for RhB for clarity. The image size was 450 × 450 µm with 256 × 256-pixel resolution. (E) Lifetime histograms from the entire FLIM images shown in (B) and (D). (F) FL and RhB lifetimes across the respective lenes on the FLIM images shown in (B) and (D) for FL and RhB, respectively.

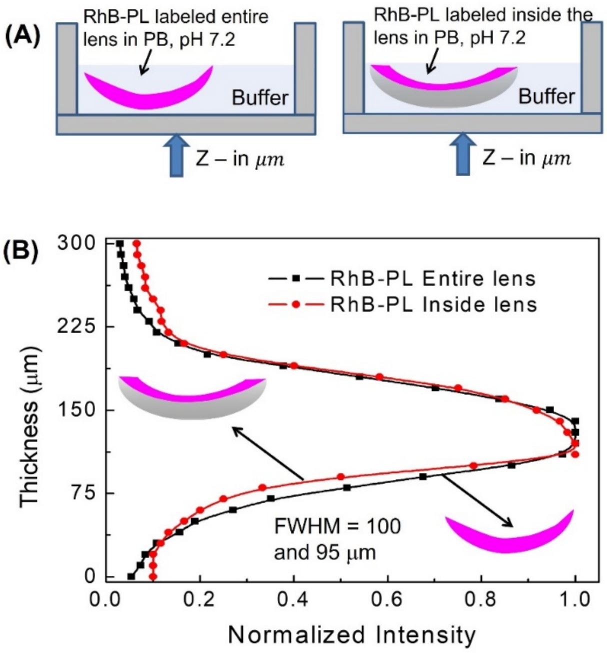

Figure S4. (A), Z-scan of emission intensity distribution of RhB-PL labeled entire or inside the Comfilcon A lenses. (B), Z-scan of emission intensity distribution of RhB-PL labeled entire or inside the Comfilcon A lenses. λex = 473 nm, 575/105 nm band-pass emission filter, 25µm pinhole, 256 × 256 pixel resolution, 450 × 450 µm image size, 20 × objective. Gray and pink lenses, respectively, for without and with RhB-PL area.
